# Regular meta-analysis of the impact of sports activities intervention on some items of the national student physical health standards for adolescents

**DOI:** 10.3389/fphys.2024.1419441

**Published:** 2024-10-24

**Authors:** Jikun Sun, Zhiyuan Sun, Jianda Kong, Xuewen Tian, Liping Wang, Qinglu Wang, Jun Xu

**Affiliations:** ^1^ Graduate School of Education, Shandong Sport University, Jinan, China; ^2^ Qufu Normal University, Qufu, China

**Keywords:** physical activity, physical health, adolescent, intervention experiment, meta-analysis

## Abstract

**Background:**

By using meta-analysis to evaluate the effects of exercise on adolescent body mass index (BMI), standing long jump, sit ups, lung capacity, sitting forward bending, 50 m running, and 800 m running, a large amount of literature will be reviewed to reveal the key role of exercise in the healthy development of adolescents. This study aims to promote the development of adolescent physical health and solve the common problem of declining physical fitness, comprehensively improve the physical fitness of adolescents, and provide decision-making support for policymakers. The research results will provide methodological references for precise and effective intervention practices, further improve adolescent physical fitness, and lay a solid physiological foundation for their comprehensive development.

**Methods:**

A literature search was conducted on China National Knowledge Infrastructure (CNKI) and PubMed, collecting randomized controlled trials (RCTs) that investigated the effects of physical activity on adolescent physical fitness according to predefined criteria. The quality of these studies was assessed, and their outcome data were analyzed using RevMan 5.4 software. The analysis encompassed 13 articles with a total of 4,633 participants, examining measures such as mean difference (MD) and heterogeneity, followed by subgroup analyses.

**Results:**

The meta-analysis revealed that physical activity had a moderate to high effect on adolescent performance in sit-ups (MD = 4.91, 95% CI = 3.41–6.41), vital capacity (MD = 120.66, 95% CI = 48.67–194.46), sit-and-reach test (MD = 0.82, 95% CI = 0.09–1.56), 50-meter dash (MD = −1.05, 95% CI = −1.48 to −0.62), and 800-meter run (MD = −18.48, 95% CI = −24.98 to −11.97). Conversely, its influence on BMI (MD = −0.31, 95% CI = −1.87 to 1.24) and standing long jump (MD = 0.1, 95% CI = 0.05–0.15) was relatively low.

**Conclusion:**

Engaging in physical activities significantly improves adolescent physical fitness. The most effective regimen involves a combination of aerobic and anaerobic exercises, with each session lasting 30 min, performed three times per week for at least 2 months. The extent of improvement in various fitness indicators, reflecting different aspects of physical fitness, is influenced by factors such as the nature of the physical activity, intervention duration, exercise frequency, and age.

**Systematic Review Registration:**

PROSPERO, identifier CRD42024568197

## 1 Introduction

Adolescence is one of the most rapidly transformative stages of human development and growth. During this period, a constellation of determinants influencing health manifest in distinctive ways and exert unique impacts on the body (World Health Organization, 2017). Students actively participating in sports can not only promote physical growth and development, but also improve the functional level of various organs and systems (LI, 2024). The latest global data show that the majority (81%) of boys and girls aged 11 to 17 engage in moderate to vigorous physical activity for less than 1 hour per day (World Health Organization, 2022). The total amount of physical activity and moderate to high-intensity physical exercise time among Chinese adolescents are relatively low, and the physical activity level of adolescents at different age groups is affected by academic pressure (Li et al., 2024). World Health Organization data indicate that globally, 81% of children and adolescents do not meet the recommended physical activity standards set by the World Health Organization, with approximately 3.2 million individuals dying from chronic diseases due to lack of physical exercise (Yang and Li, 2022). Exercise promotes health and prevents a range of diseases (Watanabe et al., 2021). Based on this, combined with the current analysis of the physical fitness status of adolescents, they need more time to engage in sports activities (Shao, 2022).

At present, adolescents commonly face substantial academic pressure, making it difficult for students to engage in adequate physical activities to strengthen their physique, a situation that is continuously worsening (Guo, 2024). The results of the National Student Physical Fitness Standards tests over the past decade in China demonstrate a declining trend in adolescent physical fitness (Wang J., 2023). Additionally, issues such as a high prevalence of vision impairment and myopia among students, an increasing rate of overweight and obesity, a decline in grip strength levels, and a drop in physical fitness among college students have also been identified (Hepartment of Physical Health and Arts Education Ministry of Education, 2021). From this, it can be seen that the trend of declining physical fitness among Chinese adolescents is increasingly related to various social factors, such as dietary habits, academic pressure, lack of exercise, etc., (Du, 2023). This downward trend not only increases the risk of chronic diseases such as cardiovascular disease, obesity, and diabetes among adolescents but also affects multiple aspects of their lives, including mental health, learning abilities, and social skills. Good physical health can mitigate the genetic predisposition to obesity in children and adolescents (Todendi et al., 2021). There is a significant positive correlation between students’ fitness behavior and physical health, and there is a certain correlation between a healthy lifestyle and physical health indicators (Wang X., 2023). Regular physical activity, including through participation in sports, helps prevent and manage non-communicable diseases such as heart disease, stroke, diabetes, as well as breast and colorectal cancer. It also aids in preventing hypertension, overweight, and obesity, while enhancing mental health and overall well-being (World Health Organization, 2023). In addition, sports activities have other benefits, such as high-intensity interval training (HIIT), which has been proved to be effective in controlling obesity and diabetes (Filipe et al., 2024); Participating in sports activities can make people show a more positive and healthy psychological and emotional state, and exercise can actively and effectively prevent and improve mental health (Wang J. et al., 2024); Physical exercise can directly or indirectly affect the occurrence of vascular aging (Wang C. et al., 2024); Sports activities can also have a positive impact on different stages of tumor diseases, potentially improving overall survival and quality of life, reducing treatment-related toxicity, and improving response to immunotherapy (Ana et al., 2024). The decline in adolescent physical fitness runs counter to the strategic needs of Healthy China (Wang et al., 2017). As is well known, the policy of promoting youth sports and health is an important guarantee for improving the physical health of young people, achieving national strategic goals such as a healthy China and a sports powerhouse (Zhu et al., 2024). Therefore, China places great emphasis on the development of adolescents’ physical fitness. The “Opinions on Building a Higher-level Public Service System for National Fitness” emphasizes fostering lifelong exercisers and ensuring that every adolescent proficiently masters at least one or more sports skills, thereby cultivating a population engaged in various sports disciplines (Xu et al., 2023).

From an article by Stojanović et al. (2023), we find that the TGfU (Teaching Games for Understanding) volleyball intervention has a notably significant effect on enhancing students’ physical fitness. However, Zhen Li’s article posits that small-sided football matches have a positive impact on students’ physical health (Li et al., 2023). Currently, there exists a multitude of perspectives on methods to enhance adolescent physical fitness, with different exercise modalities exerting direct and profound influences on their physical qualities. Existing research fails to definitively identify the most efficacious mode of exercise. To address the issue of improving adolescent physical fitness, this paper integrates a larger dataset, compiling the test results of over four thousand individuals, and conducts a more thorough analysis of the sample. The aim is to discern, through comparative analysis, which type of exercise is most effective in boosting adolescent physical fitness, as well as how to effectively combine various exercise approaches to promote their healthy growth and development.

## 2 Research methodology

### 2.1 Literature retrieval strategy

Two researchers selected CNKI, PubMed, and Web of Science databases for literature search. The search time was to build the database until December 2023, and the last search date was 23 December2023. The relevant information was found through the included references, and the unpublished literature was not searched. See Table 1 for the search keywords. Each group of search topic words is connected with “OR”, “AND”, and Boolean operation. In addition, the references of the included literature were traced back by a manual search to ensure the comprehensiveness of the included literature.

**TABLE 1:** The search terms of the literature included in this study.

Group	Subject word
1	Exercise, physical activity, aerobic exercise, physical training, running, resistance exercise, physical training
2	Physical fitness, physique, health, national physical health standards, BMI, standing long jump, sit-up, lung capacity, sitting body forward flexion, 50-meter running, 800-meter running
3	Intervention experiment, intervention studies, randomized controlled trial

### 2.2 Inclusion and exclusion criteria

Literature type: The experimental design was a randomized controlled trial (RCT), regardless of whether it was blind or not. Study inclusion criteria: 1) Study subjects: Chinese adolescents were selected as the study subjects, and the study subjects were required to be healthy and disease-free. 2) Intervention measures: Exercise intervention was the only intervention measure in the experimental group, with no restrictions on exercise methods, and the control group received routine physical education courses in the school. 3) Outcome indicators: All or part of the indicators (standing long jump, sit ups, lung capacity, sitting forward bending, BMI, 50 m run, 800 m run) tested by the National Physical Fitness Monitoring Standards were used as the outcome indicators. 4) Study design: This study was published in both Chinese and English. A randomized controlled trial was conducted, and there was no significant difference between the experimental group and the control group. Exclusion criteria: 1) studies unrelated to the meta-analysis topic. 2) incomplete outcome indicator data, insufficient data to calculate the mean difference (MD) score after intervention. 3) duplicate or overlapping articles; 4) Review, editorial, animal experiments, or thesis.

### 2.3 Literature screening and data information extraction

Firstly, the collected literature will be uniformly imported into the literature management software “NoteExpress” and screened: first, duplicate literature in the database will be excluded, and then two researchers will further screen the entire text according to the inclusion and exclusion criteria. Two researchers will compare the included literature, and if the results are different, a third party will decide whether to include it. Independent extraction of literature that meets the criteria will be carried out, including: literature title, first author, publication time, subject age, sample size, sports activity content, exercise frequency, exercise cycle, intervention duration, and outcome indicators.

### 2.4 Risk assessment of bias included in literature

The bias risk assessment included in the literature was conducted using Cochrane Collaboration’s RCT bias assessment tool (Higgins et al., 2011). Evaluate: 1) the generation of random sequences; 2) Allocation hidden; 3) Blind method between implementers and participants; 4) Blind method for outcome evaluation; 5) The completeness of the result data; 6) Selective reporting; 7) Other sources of bias. The risk of bias will be independently evaluated by two researchers. If there is any disagreement, it will be resolved through negotiation or discussion with the third researcher. Each indicator will be judged based on low bias risk, bias uncertainty, and high bias risk. Based on the judgment results, the quality of the included literature will be divided into three levels: 1) If four or more items are met with low risk, it will be rated as A level; 2) Satisfying 2 or 3 low risk items, rated as B level; 3) Satisfying 1 or no low risk item, rated as C-level.

### 2.5 Statistical methods

Revman 5.4 was used to statistically analyze the outcome indicators of the included literature. Firstly, heterogeneity testing was performed on the literature, using the Homogeneity test (with a criterion of a = 0.1), i.e., the X2 test. If *P* < a, it indicates heterogeneity between the studies; On the contrary, the studies are homogeneous. I2 is used to quantitatively evaluate the heterogeneity between studies, and I2 statistics are used to test the level of heterogeneity between studies. According to the Cochrane Handbook evaluation criteria, when I2 = 0, it is considered that there is no heterogeneity between studies; When I2 < 50%, it is considered that there is low heterogeneity between the studies, which is acceptable. A fixed effects model is used for analysis. When I2 > 50%, there is significant heterogeneity between the studies, and a random effects model is used for analysis. If there is high heterogeneity between the studies, subgroup analysis is conducted based on the sources of heterogeneity generated. Funnel plots and Egger tests are used to analyze the impact of publication bias. The test units of the results indicators included in the literature are consistent, so the mean difference is used as the effect size, calculated with a 95% confidence interval, and P < 0.05 is considered significant for the difference.

## 3 Results

### 3.1 Literature search results

**FIGURE 1:**
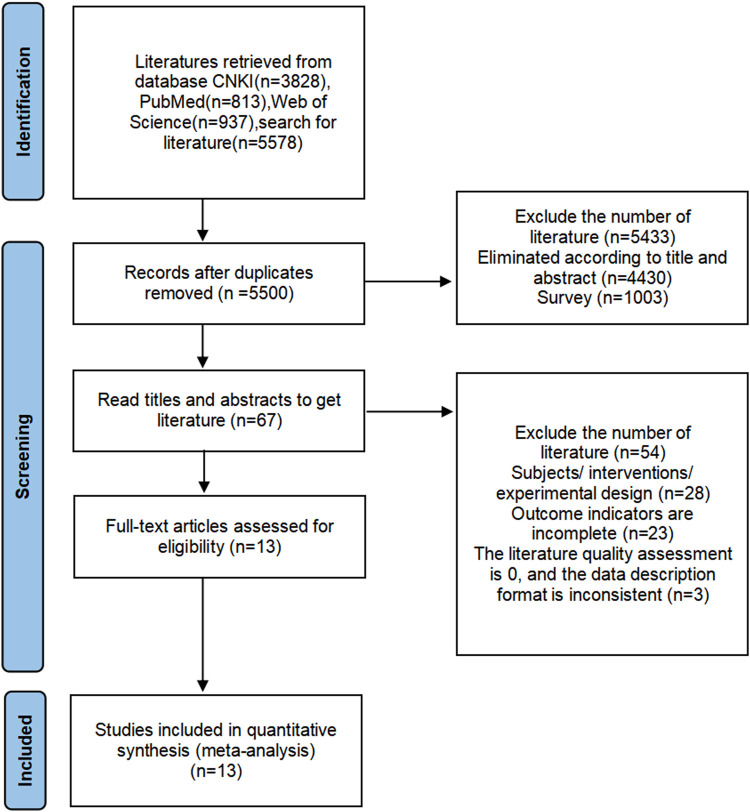
Literature selection flow diagram..

As shown in Figure 1, according to the formulated search strategy, a total of 5,578 potentially relevant articles were retrieved, out of which 50 were published in international journals. Upon applying the established inclusion and exclusion criteria, 13 articles were ultimately selected for inclusion in the analysis, comprising 9 domestically published studies and 4 studies from overseas sources.

### 3.2 Basic characteristics of inclusion in literature

This study included a total of 13 articles, with a total sample size of 4,633 people (approximately 48% of males). All participants were adolescents, and both the experimental and control groups reported sample sizes, with 2,008 people in the experimental group and 2,625 people in the control group. Intervention content for the experimental group: 9 articles used aerobic exercise, 4 articles used anaerobic exercise, 7 articles used exercise frequency ≤3 days/week, 1 article had exercise frequency > 3 days/week, 5 articles did not specify exercise frequency, 5 articles had exercise cycle ≤10 weeks, 5 articles had exercise cycle > 10 weeks, 3 articles did not specify exercise cycle, and 5 articles did not specify exercise cycle. Time < 40 min/time, exercise time ≥40 min/time in 4 articles, exercise time not specified in 3 articles, and intervention measures in the control group mostly focused on routine physical activities. The detailed characteristics of the included literature are shown in Table 2.

**TABLE 2:** Baseline characteristics of the thirtee included studies.

First author (Year)	Age (years)	Sample size		Content of sports activities	Frequency (times/week)	Time (min/week)	Intervention duration	Outcome indicators
		Intervention group	Control group					
Zhu (2023)	9∼12	50	50	aerobic exercise	3	90	12	BMI; 50 m run
Zheng and He (2023)	13∼16	128	128	aerobic exercise	5	100	18	Lung capacity; 800 m run; 1000 m run
Jiang et al. (2022)	18∼20	135	135	aerobic exercise	NA	NA	15	Standing long jump; sitting forward flexion; 1 min sit-up; 50 m run
Jing (2022)	8∼9	30	30	aerobic exercise	3–4	120	10	Standing long jump; lung capacity, sitting forward flexion; BMI; 1 min sit-up
Jing (2022)	8∼9	30	30	aerobic exercise	3–4	120	10	Standing long jump; lung capacity, sitting position; BMI; 1-minute sit-up
Yan (2022)	9∼10	30	30	aerobic exercise	3	90	16	Standing long jump; sitting forward bending; kneeling; sit-ups
Xue (2021)	14∼15	35	35	aerobic exercise	2	15	8	Lung vital capacity; 800 m; 1000 m
Xue (2021)	14∼15	35	35	aerobic exercise	2	15	8	Lung vital capacity; 800 m; 1000 m
Wang (2019)	19∼23	200; 200	200	aerobic exercise	NA	NA		Standing long jump; lung capacity, sitting forward flexion; BMI; sit-ups; 50 m run; 800 m run
Yang et al. (2012)	17∼18	230	510	anaerobic exercise	3	120	10	Standing long jump; sitting body forward flexion; pull-up
Yang et al. (2012)	14∼15	200	560	anaerobic exercise	3	120	10	standing long jump; BMI
Yang et al. (2012)	10∼11	314	338	anaerobic exercise	3	120	10	vital capacity
Yang et al. (2012)	9∼10	338	298	anaerobic exercise	3	120	10	Standing long jump; lung capacity
Song (2011)		4	4	anaerobic exercise	NA	40	16	standing long jump
Bulca et al. (2022)	11∼12	28	34	aerobic exercise	3	NA	8	sit-up; situp; abdominal curl
Griban et al. (2021)	19∼22	60	52	aerobic exercise	NA	240	NA	Standing long jump; sit-ups
Griban et al. (2021)	19∼22	79	81	aerobic exercise	NA	240	NA	Standing long jump; sit-ups
Mayorga-Vega et al. (2012)	11.1	35	34	anaerobic exercise	2	100	8	Standing long jump; sit-ups
Zhamardiy et al. (2020)	18∼22	47	41	anaerobic exercise	NA	NA	NA	Standing long jump; sit-ups

Note: & Group for men, # Group for women, Unlabeled is a mixed grouping; Dan Yang (1) (2) (2) (3) (4) Four groups in the same document.

### 3.3 Risk bias assessment for inclusion in literature

**FIGURE 2:**
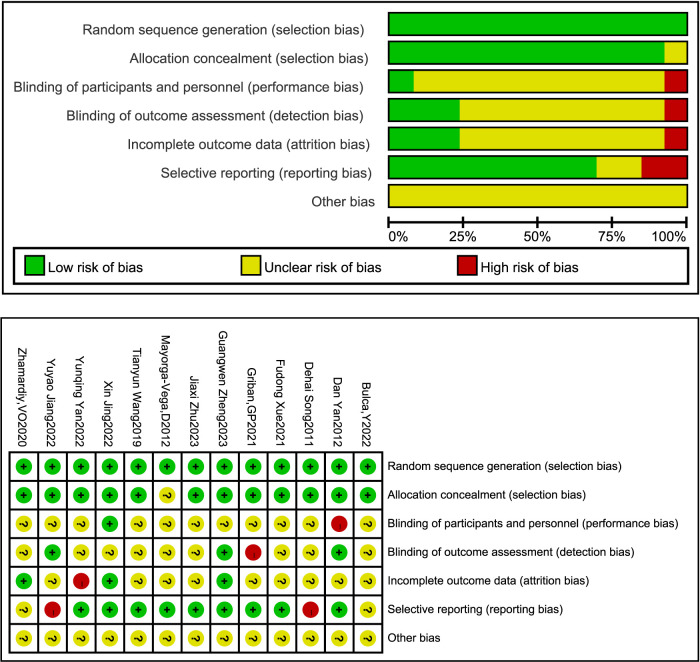
Analysis of the risk of bias in accordance with the cochrane collaboration guidelines.

The two authors used the Cochrane bias risk assessment tool to evaluate the quality of the aforementioned literature. If there were differences, they were handed over to the third author for evaluation, and the final evaluation result of the literature quality was obtained. According to the evaluation criteria of literature quality, as shown in Figure 2, there were 3 low-risk literature that met 4 or more items, and 10 low-risk literature that met 2-3 items. The evaluation results were 3 A-level and 10 B-level literature quality.

### 3.4 Meta analysis results

#### 3.4.1 The influence of sports activities intervention on the performance of teenagers in standing long jump

**FIGURE 3:**
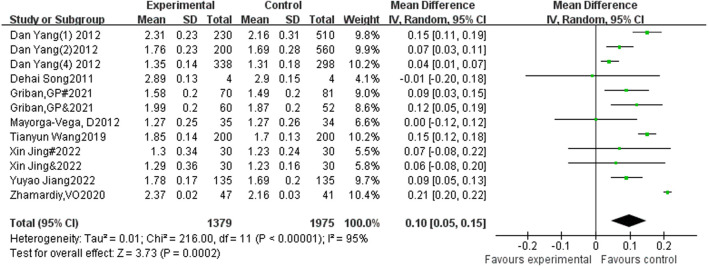
Forest plot of the effect of physical activity intervention on adolescent standing long jump performance.

A total of 12 studies included standing long jump as an indicator, with 1,379 participants in the experimental group and 1,975 participants in the control group. Heterogeneity testing showed that there was significant heterogeneity among the 12 studies. I^2^ = 95% > 50%, *P* < 0.00001, so a random effects model was used to merge the results. After merging, the effective stress MD = 0.1, 95% CI = 0.05∼0.15, *P* < 0.01 (See Figure 3).

#### 3.4.2 The impact of sports intervention on sit up performance among adolescents

**FIGURE 4:**
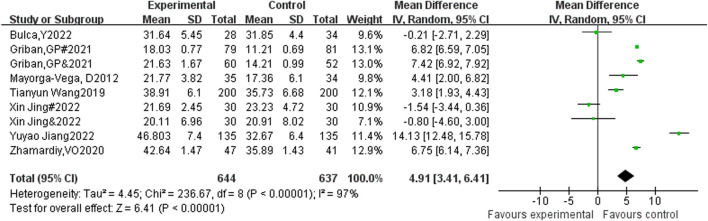
Forest plot of the effect of physical activity intervention on adolescent sit-up performance.

A total of 9 studies included sit ups as an indicator, with 644 participants in the experimental group and 637 participants in the control group. Heterogeneity testing showed significant heterogeneity among the 9 studies. I^2^ = 97% > 50%, *P* < 0.00001, so a random effects model was used to merge the results. After merging, the effective response MD = 4.91, 95% CI = 3.41∼6.41, *P* < 0.00001 (See Figure 4).

#### 3.4.3 The impact of sports intervention on adolescent lung capacity performance

**FIGURE 5:**
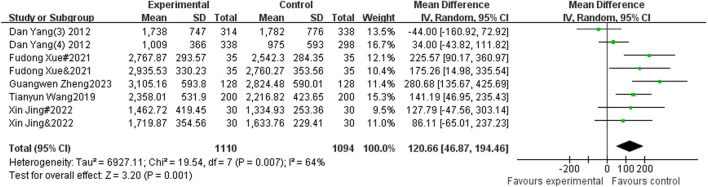
Forest plot of the effect of physical activity intervention on adolescent pulmonary performance.

A total of 5 studies included sitting forward flexion as an indicator, with 1,110 participants in the experimental group and 1,094 participants in the control group. Heterogeneity testing showed that there was significant heterogeneity among the 5 studies. I^2^ = 64% > 50%, P = 0.007, so a random effects model was used to merge the results. After merging, the effective stress MD = 120.66, 95% CI = 48.67∼194.46, P = 0.001 (See Figure 5).

#### 3.4.4 The effect of sports activities intervention on sitting forward bending performance of adolescents

**FIGURE 6:**
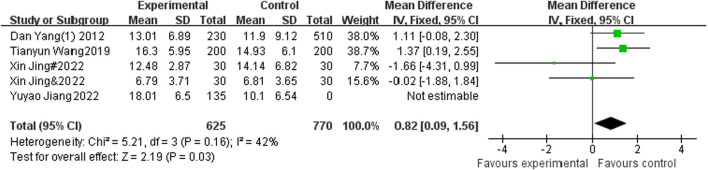
Forest plot of the effect of physical activity intervention on adolescent Sit forward performance.

A total of 5 studies included sitting forward flexion as an indicator, with 625 participants in the experimental group and 770 participants in the control group. Heterogeneity testing showed that there was small heterogeneity among the 5 studies. I^2^ = 42% < 50%, *P* = 0.16, so a fixed effects model was used to merge the results. After merging, the effective stress MD = 0.82, 95% CI = 0.09–1.56, *P* = 0.03 (See Figure 6).

#### 3.4.5 The impact of sports intervention on adolescent BMI scores

**FIGURE 7:**
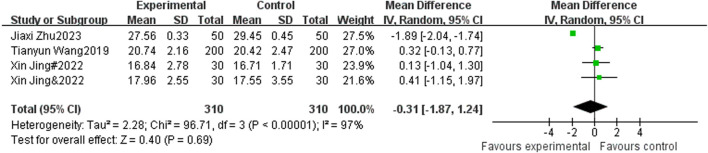
Forest plot of the effect of physical activity intervention on adolescent BMI performance.

There were 4 studies that included BMI as an indicator, with 310 participants in the experimental group and 310 participants in the control group. Heterogeneity testing showed significant heterogeneity among the 4 studies. I^2^ = 97% < 50%, *P* < 0.00001, so a random effects model was used to merge the results. After merging, the effective stress MD = −0.31, 95% CI = −1.87∼1.24, *P* = 0.69 (See Figure 7).

#### 3.4.6 The influence of sports activities intervention on the performance of teenagers in 50 m running

**FIGURE 8:**
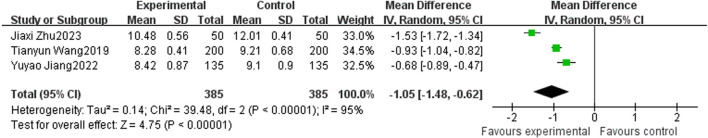
Forest plot of the effect of physical activity intervention on adolescent Run 50 m performance.

Three studies included 50 m running as an indicator, with 385 participants in the experimental group and 385 participants in the control group. Heterogeneity testing showed significant heterogeneity among the three studies. I^2^ = 95% < 50%, *P* < 0.00001, so a random effects model was used to merge the results. After merging, the effective stress MD = −1.05, 95% CI = −1.48∼−0.62, *P* < 0.00001 (See Figure 8).

#### 3.4.7 The influence of sports activities intervention on the performance of teenagers in 800 m running

**FIGURE 9:**
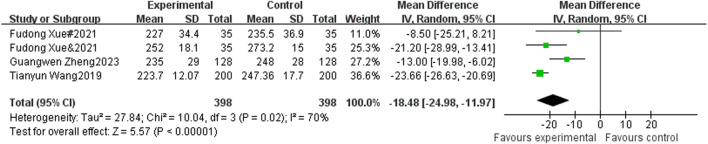
Forest plot of the effect of physical activity intervention on adolescent Run 800 m performance.

A total of 4 studies included 800 m running as an indicator, with 398 participants in the experimental group and 398 participants in the control group. Heterogeneity testing showed that there was significant heterogeneity among the 4 studies. I^2^ = 70% < 50%, *P* = 0.02, so a random effects model was used to merge the results. After merging, the effective stress MD = −18.48, 95% CI = −24.98∼−11.97, *P* < 0.00001 (See Figure 9).

### 3.5 Sensitivity analysis and publication bias of the impact of sports intervention on the standing long jump performance of adolescents

In sensitivity analysis, the combined results of the meta-analysis using the random effects model are consistent with the combined results of the fixed effects model or the main results of the study with the lowest literature quality score removed, indicating that the combined effects of exercise intervention on cognitive function (MD) are stable. To investigate hair bias, funnel plots are usually performed on meta-analyses of more than 10 studies and analyzed. It can be seen that all studies have a good symmetrical distribution; Then, Egger test (Table 3) was performed, with *P* = 0.024 < 0.05, indicating no significant publication bias.

**TABLE 3:** Egger test results.

Std_Eff	Cofe	Std.Eff	t	*p* > |t|	(95%Conf.Interval)
Slope	0.2,094,037	0.0225,392	9.29	0.000	(0.1,591,833,0.2,596,242)
Bias	−4.006592	1.504,894	−2.66	0.024	(-7.359,705,-.6,534,799)

### 3.6 Subgroup analysis of the impact of sports intervention on the standing long jump performance of adolescents

#### 3.6.1 The influence of different sports methods on the performance of teenagers in standing long jump

**FIGURE 10:**
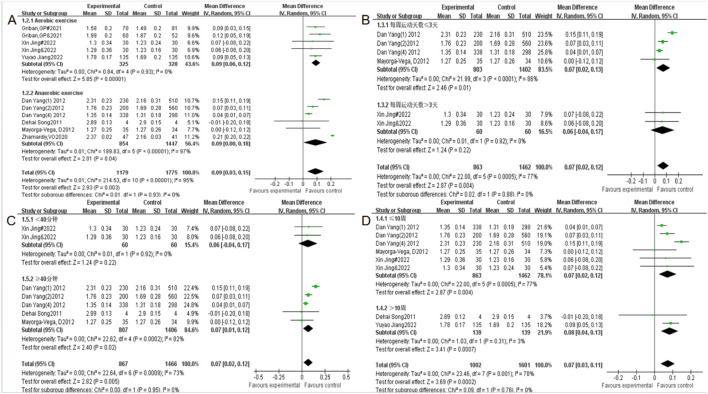
Forest plot of the effect of a physical activity intervention on adolescent standing long jump performance: **(A)** Exercise form **(B)** Exercise frequency **(C)** Exercise time **(D)** Exercise duration.

This group included a total of 11 studies with 2,954 participants (see Table 3). The exercise methods were divided into two forms: aerobic exercise and anaerobic exercise for subgroup analysis (Figure 10A). The difference in effect size between the two groups was close to high heterogeneity (I^2^ = 95%), indicating that the intervention content had a significant regulatory effect on the intervention effect. Among them, heterogeneity testing in the aerobic exercise group showed X^2^ = 0.84, I^2^ = 0%, *P* = 0.93, combined effect size MD = 0.06, 95% CI: 0.06–0.12, *P* < 0.00001, no statistically significant difference; Heterogeneity test of anaerobic exercise group shows X^2^ = 189.83, I^2^ = 97%, *P* < 0.00001, combined effect size MD = 0.09, 95% CI: 0∼0.18, *P* = 0.04, the difference is statistically significant. It can be seen that aerobic exercise has the most significant effect size.

#### 3.6.2 The influence of exercise frequency on the performance of teenagers in standing long jump

This group included a total of 6 studies and 2,325 subjects (see Table 3). The exercise frequency was divided into two groups: exercise days ≤3 days per week (including 3 days) and exercise days >3 days per week. The two groups had high heterogeneity in effect size (I^2^ = 77%), indicating that exercise frequency has a significant regulatory effect on intervention effectiveness (Figure 10B). The heterogeneity test of exercise days ≤3 days per week showed: X^2^ = 21.99, I^2^ = 86%, *P* < 0.0001, combined effect size MD = 0.07, 95% CI: 0.02–0.13, *P* = 0.01, the difference is statistically significant. Heterogeneity test for exercise >3 days per week shows: X^2^ = 0.01, I^2^ = 0%, *P* = 0.92, combined effect size MD = 0.06, 95% CI: −0.04∼0.17, *P* = 0.22, no statistically significant difference.

#### 3.6.3 The influence of exercise cycle on the performance of teenagers in standing long jump

This group included a total of 8 studies with 2,603 participants (see Table 3), and divided the exercise cycle into ≤10 weeks (including 10 weeks) and >10 weeks. The difference in effect size between the two groups was close to high heterogeneity (I^2^ = 70%), indicating that the exercise cycle has a significant moderating effect on the intervention effect (Figure 10C). The heterogeneity test of adolescent standing long jump performance for exercise duration ≤10 weeks showed: X^2^ = 22, I^2^ = 77%, *P* = 0.0005, combined effect size MD = 0.07, 95% CI: 0.02–0.12, *P* = 0.004, the difference is statistically significant; Heterogeneity test of standing long jump performance in adolescents with exercise duration greater than 10 weeks shows X^2^ = 1.03, I^2^ = 3%, *P* = 0.31, combined effect size MD = 0.08, 95% CI: 0.04–0.13, *P* = 0.0007, the difference is statistically significant, and the effect size is most significant for exercise cycles >10 weeks.

#### 3.6.4 The influence of exercise time on the performance of teenagers in standing long jump

This group included a total of 7 studies with 2,393 participants (see Table 4). The exercise time was divided into single exercise time <40 min and single exercise time ≥40 min (including 40 min). The two groups showed high heterogeneity in effect size differences (I^2^ = 73%), indicating that exercise time has a significant regulatory effect on intervention effects (Figure 10D). Heterogeneity tests for single exercise time <40 min showed: X^2^ = 0.01, I^2^ = 0%, *P* = 0.92, combined effect size MD = 0.06, 95% CI -0.04–0.17, *P* = 0.22, no statistically significant difference, heterogeneity test for single exercise ≥40 min showed: X^2^ = 22.62, I^2^ = 82%, *P* = 0.0002, combined effect size MD = 0.07, 95% CI: 001∼0.12, *P* = 0.02, the difference is statistically significant.

**TABLE 4:** The effect of exercise intervention on standing long jump performance in adolescents.

Regulated variable	Homogeneity-testing			Category	MD	95%CI	Two-tail test		Documents quantity	Sample size
	X^2^	*P*	I^2^/%				Z	*P*		
Means of intervention	214.53	<0.00001	95	Aerobic exercise	0.09	(0.06, 0.12)	5.85	<0.00001	5	653
				Anaerobic exercise	0.09	(0, 0.18)	2.01	0.04	6	2,301
Frequency of intervention	22	0.0005	77	≤3 times/week	0.07	(0.02, 0.13)	2.46	0.01	4	2,205
				>3 times/week	0.06	(-0.04, 0.17)	1.24	0.22	2	120
Intervention cycle	23.46	0.001	70	≤10 weeks	0.07	(0.02, 0.12)	2.87	0.004	6	2,325
				>10 weeks	0.08	(0.04, 0.13)	3.41	0.0007	2	278
Intervention duration	22.64	0.0009	73	<40 min/times	0.06	(-0.04, 0.17)	1.24	0.22	2	120
				≥40 min/times	0.07	(0.02, 0.12)	2.82	0.005	5	2,273

### 3.7 Subgroup analysis of the impact of sports intervention on sit up performance among adolescents

#### 3.7.1 The influence of different exercise methods on the performance of sit ups in adolescents

**FIGURE 11:**
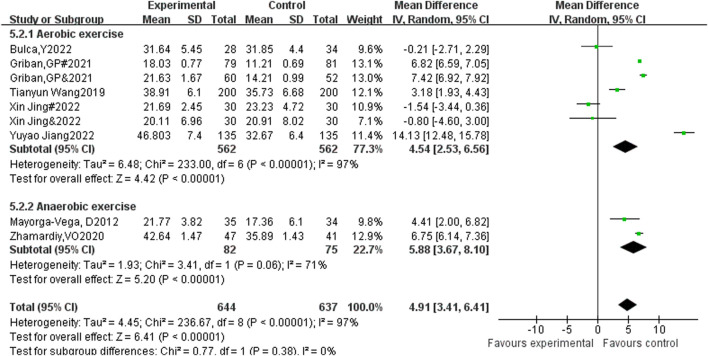
Forest plot of the effect of a physical activity intervention on adolescent sit-up performance.

This group included a total of 9 studies, with 1,281 participants. The exercise methods were divided into two forms: aerobic exercise and anaerobic exercise for subgroup analysis (Figure 11). The difference in effect size between the two groups was close to high heterogeneity (I^2^ = 97%), indicating that the intervention content had a significant regulatory effect on the intervention effect. Among them, the heterogeneity test of the aerobic exercise group showed X^2^ = 233, I^2^ = 97%, *P* < 0.00001, combined effect size MD = 4.54, 95% CI: 2.53–6.56, *P* < 0.00001, the difference is statistically significant; Heterogeneity test of anaerobic exercise group shows X^2^ = 3.41, I^2^ = 71%, *P* = 0.06, combined effect size MD = 5.88, 95% CI: 3.67–8.1, *P* < 0.00001, the difference is statistically significant. It can be seen that aerobic exercise has the most significant effect size.

## 4 Discuss

Numerous studies have already demonstrated the efficacy of physical exercise in enhancing the physical fitness of adolescents; however, the precise magnitude of this effect remains unclear. The present meta-analysis of 13 included articles reveals that, compared to control groups, physical activity interventions generally exert a positive influence on the performance of adolescents in standing long jump, sit-ups, vital capacity, sit-and-reach test, body mass index (BMI), 50-meter dash, and 800-meter run. Nonetheless, improvements in certain performance metrics were found to be statistically nonsignificant.

Standing long jump is an important sports event for cultivating students’ physical fitness (Yang, 2021), It plays an important role in improving students’ speed, strength, and coordination abilities (Shang, 2023). The meta-analysis results of this study showed that among the 12 studies included, including standing long jump, one study was statistically insignificant, with a combined MD value of 0.1. In the study, the sports cycle is mostly around 10 weeks, which may be due to considerations for the adolescent semester. Generally, a semester for adolescent students lasts for 16 weeks. Excluding pre-test, post test, and other preparatory activities, a 10 weeks intervention exercise cycle is more appropriate. From the perspective of intervention period, the effect of exercise intervention for more than 10 weeks was greater than that for 10 weeks and below; From the perspective of intervention time, exercise intervention with a duration of 40 min or more has a better effect than exercise intervention with a duration of less than 40 min; From the perspective of intervention frequency, the effect of a frequency of exercise days ≤3 days per week is better than a frequency of exercise days above 3 days. It can be seen that training with longer cycles and longer single training times will have better effects. However, from the perspective of exercise frequency, we found that it is not necessarily better to train more frequently per week. Because longer rest time can enhance people’s muscle strength (Brad et al., 2024), Post-exercise recovery is far more than just “rest”; it is a vital process of recharging the body, preparing it for the next round of exertion (Cao, 2023). In summary, physical activity interventions with longer durations (over 10 weeks) and longer training times (at least 40 min per session) have better effects. In addition, exercising no more than three times a week is more effective than frequent high-intensity training. These conclusions remind us that a scientifically reasonable training plan should consider the importance of training continuity and recovery period, and avoid the negative effects of overtraining.

Sitting forward bending is an indicator of flexibility reflected in the national student physical health standards (Sun and Sun, 2022), Pulmonary capacity is one of the indicators in lung function measurement. The meta-analysis results of this study showed that all 5 studies including sitting forward flexion had statistical significance, and the combined intervention effect was high. There were a total of 8 studies including pulmonary capacity, all of which had statistical significance, and the combined intervention effect was high. At the same time, when conducting subgroup analysis based on the sports content studied, the MD values of studies with aerobic exercise as the intervention method were much higher than those with anaerobic exercise. Therefore, it can be seen that performing aerobic exercise alone or combining resistance exercise with aerobic exercise, but not resistance exercise alone, can improve the risk of composite cardiovascular disease (Duck-Chul et al., 2024). And aerobic training is also associated with reduced visceral fat in the abdomen, which may be beneficial for the cardiovascular metabolic health of obese individuals (Jean-Michel et al., 2023). So, when designing sports activities, combining aerobic and anaerobic exercise is the optimal choice. For example, aerobic exercise has shown higher efficacy in improving cardiovascular function; In terms of enhancing abdominal muscle endurance, anaerobic exercise is recommended. This indicates that when designing youth sports activity plans, the characteristics of different types of sports should be taken into account to achieve the goal of comprehensively improving the physical fitness of young people.

The purpose of sit ups is to test abdominal muscle endurance (Wang and Shang, 2022). The meta-analysis results of this study showed that all 9 studies containing sit ups were statistically significant and had high intervention effects after merging. At the same time, when conducting subgroup analysis based on the physical activity content of the study, it was found that the MD value of anaerobic exercise was slightly higher than that of aerobic exercise, because sit ups are anaerobic exercise (Yang et al., 2024). However, we found that Yuyao Jiang’s body function training also had a good effect on improving sit up performance. Therefore, we can combine anaerobic exercise training with body function training to improve sit up performance.

Body mass index (BMI) is an important indicator of human health (Xiang et al., 2024). And the 50 m run and 800 m run correspond to speed (Han, 2022) and endurance (QIao, 2022), respectively. The meta-analysis results of this study showed that all 8 articles included in the 3 outcome indicators had statistical significance, and the combined effect was good.

In addition, we can also explore the optimal exercise combination mode, such as comparing the effects of anaerobic exercise and aerobic exercise. We found that aerobic exercise has a significant effect on improving lung capacity and sitting forward bending, indicating that aerobic exercise can effectively improve the cardiovascular function and flexibility of adolescents; Anaerobic exercise is slightly better than aerobic exercise in improving sit up performance, because sit ups mainly test abdominal muscle endurance and belong to the category of anaerobic exercise. In addition, we found that Yuyao Jiang’s physical function training had a good effect on improving sit up performance, indicating that functional training can be added to anaerobic exercise training.

Through discussion, we found that by participating in sports activities, teenagers can improve their physical function and develop healthy lifestyle habits. This is of great significance for preventing chronic diseases such as obesity and cardiovascular disease. At the same time, the results of this study can provide important reference for policymakers when planning school physical education curriculum and public health strategies. By drawing on this discussion, we can better formulate sports policies that meet the growth needs of young people and promote their healthy development. In addition, schools and families can encourage young people to actively participate in sports activities based on research recommendations, and focus on the quality of activities rather than quantity. Through appropriate exercise interventions, help children improve their physical fitness while enjoying the fun of sports, laying a solid foundation for the future.

## 5 Conclusion

Based on the meta-analysis results of the seven physical fitness outcome indicators mentioned above, it can be concluded that sports content, cycle, single time, and frequency are important influencing factors for the intervention effect of adolescent sports activities.

Regular physical exercise has shown moderate to high effects in improving adolescent sit ups, lung capacity, flexibility (sitting forward), short distance speed (50 m run), and long-distance endurance (800 m run). However, for BMI index and standing long jump, the influence of sports activities is relatively low.

Further research has found that the most effective exercise intervention plan should be a combination of aerobic and anaerobic exercise, and it is recommended to exercise for no less than 30 min each time, at least three times a week, and for a duration of at least 2 months. This training frequency and duration can be accepted by most teenagers, and can leave enough time for recovery after exercise to achieve the best training results.

In addition to the above factors, the sensitive period of adolescent physical fitness development is also an important factor affecting the effectiveness of intervention. For example, the speed sensitive period is between the ages of 14–16; The period of strength sensitivity is between 13 and 17 years old; The endurance sensitive period is from 16 to 18 years old. So when conducting research grouping. It is essential to fully consider the age characteristics and developmental stages of adolescents.

In summary, this study confirms the importance of physical activity in improving adolescent health and provides strong evidence to support the development of more scientific and reasonable exercise intervention plans. For sports educators, sports coaches, parents, and policymakers, these findings will help guide the planning and implementation of youth sports activities, thereby promoting the healthy growth of young people and contributing to the construction of a “Healthy China”.

## Data Availability

The original contributions presented in the study are included in the article/supplementary material, further inquiries can be directed to the corresponding authors.
